# Stroke Care in South Asia – Identifying Gaps for Future Action

**DOI:** 10.5334/gh.1351

**Published:** 2024-08-22

**Authors:** Shiva Raj Mishra, Kanghui Wei, Edel O’Hagan, Vishnu Khanal, Maarit A. Laaksonen, Richard I. Lindley

**Affiliations:** 1 NHMRC Clinical Trials Centre, University of Sydney, Australia; 2 Westmead Applied Research Centre, University of Sydney, Australia; 3 Nepal Development Society, Bharatpur-6, Chitwan, Nepal; 4 Menzies School of Health Research, Charles Darwin University, Alice Springs, NT, Australia; 5 School of Public Health, University of Sydney, Australia

**Keywords:** stroke, epidemiology, risk factor control, blood pressure, South Asia

## Abstract

Stroke causes around 730,000 deaths in South Asia, nearly half of stroke-related deaths in developing countries. This highlights the need to address health system responses, considering poverty, service quality, and availability. The article identifies four key challenges in stroke management and rehabilitation in South Asia, emphasizing long-term monitoring, risk factor control, and community surveillance, drawing on experiences from Nepal.

Stroke contributes to nearly 730,000 deaths in South Asia which accounts for nearly 50% of stroke mortality in developing countries (1). In particular, the region has a higher proportion of stroke due to haemorrhagic stroke (2). Stroke related mortality and disability is increasing due to population aging and growth in risk factor burden (1,3). This emphasizes the importance of studying health system responses to these growing risk factors and the resulting disease burden in South Asia, and how this is intertwined with poverty, service quality, and availability in the region. In this piece, we discuss four key challenges to stroke management and rehabilitation in South Asia and highlight the importance of long-term stroke monitoring, risk factor control, and surveillance at the community, drawing experiences from our work on hypertension management in Nepal (4,5,6).

First, sustained focus on risk factor management is needed in South Asia. Given that stroke is largely preventable, controlling risk factors through lifestyle modification could prevent up to 55% of stroke risk (7) and lead to further benefits obtained through blood pressure screening (8). The American Heart Association guidelines (9) highlight the importance of risk factor management (smoking, physical activity, being overweight, blood pressure control, diabetes, dyslipidemia) as the key to primary prevention of stroke. Of these risk factors, effective blood pressure control represents the potentially highest-impact intervention for reducing stroke.

Second, the devolution and decentralization of health services in countries like Nepal has increased pressure on already overstretched public health care services which need improvement in service delivery and efficiency (1). Early diagnosis and treatment of stroke is currently inaccessible as the health systems largely remain unprepared (10). Likewise, secondary prevention and rehabilitation post stroke is also lacking, the reasons for which are multidimensional. Rehabilitation services are primarily provided by the private sector in Nepal, India, and Bangladesh (11). As a result, the lack of formal (public or private) insurance coverage for a large segment of the population results in high out-of-pocket expenditures for stroke care. This can lead to catastrophic financial hardship driving incidental poverty in affected regions, particularly for individuals requiring long-term care (12). In low-income settings, rehabilitation services are not yet fully established as a distinct discipline. Care often focuses primarily on physiotherapy, and the long-term benefits of comprehensive rehabilitation programs, including workforce training, are not yet widely recognized.

Third, greater efforts are needed to develop surveillance capacity for ongoing and systematic collection of data for planning, implementation, and evaluation of stroke services. Most studies on stroke in South Asia are researcher driven rather than driven by institutions which either have low or no funding. Furthermore, these studies are restricted in terms of geography and have varying methodological limitations. In *The Lancet*, Kalkonde and colleagues reported that only India and Thailand have population-based stroke registries to monitor stroke-mortality. In India, the National Stroke Registry Program (NSRP) has population-based stroke registries in place covering a population of ~12 million (3). To address the paucity of nationally representative stroke data, national health information systems (HMIS) including hospital-based registries can be capitalized on to make stroke types, risk factors, clinical events, and survivorship data available, thus increasing the usefulness of available data (3). Furthermore, systematizing data collection with a strong push towards digital systems and electronic records will allow inter-country and center comparison which will help to monitor progress in stroke care and rehabilitation – as well as identify gaps and inequities in care across the region.

Fourth, the generally low volume of clinical trials conducted in South Asia is an important barrier for advancement in stroke care in this region. This is due to a lack of clinical trial infrastructure, a lack of funding, and a weak training capacity and mentorship for future trialists. Our brief review of clinical trial activity in South Asia using World Health Organization’s International Clinical Trial Registry Platform (ICTRP) found that only 446 of trials were conducted in South Asia region (from 2000 to 2023), with the highest number of stroke trials in India (n = 221), Pakistan (n = 192), Sri Lanka (n = 17), Bangladesh (n = 7), Nepal (n = 6), and one trial each in Afghanistan, Bhutan, and Maldives (n = 1). The sheer paucity of clinical trials in the South Asia region is particularly concerning and needs focus on improving clinical trial capacity in the region. However, newer initiatives such as the INSTRuCT stroke trial network in India funded by the Indian Council of Medical Research are starting to address this issue. Six trials have been approved (two completed and four ongoing) which aim at addressing data paucity, paving a new horizon for stroke research (13).

Despite challenges, there are some palpable signs for improvement in stroke management, which is already happening through increasing penetration of mobile based technology which is making development and expansion of low-cost telestroke models of care possible in the South Asia region. Telestroke is a model of care that aims to provide acute ischemic stroke treatment using computerized axial tomographic scan data remotely using smartphones. This approach holds promise for regions like Indonesia, Bhutan, Afghanistan, and Nepal where challenging geographical landscapes limit access to stroke specialists (11). We expect that increasing penetration of smartphone devices (with comparable effectiveness to medical monitors) and decreasing internet costs will boost telestroke services expansion in the region. Furthermore, addressing low health literacy and language barriers through awareness and translation services will help to ensure that these services are available and remain useful across cities and international borders.

Given the massive burden of stroke in South Asia, innovative approaches are needed to target modifiable risk factors. Community-based blood pressure screenings using existing networks of community health workers can alleviate pressure on stretched health systems, and free up time for acute stroke care and rehabilitation services (4,5,6). Evidence from Nepal’s May Measurement Month, a nationwide blood pressure screening campaign since 2017, exemplifies the effectiveness of such programs (6,14). Of 5,972 participants screened in 2017, 24.4% were hypertensive, 37.6% were on antihypertensive medication and of these 54.8% were had controlled blood pressure (6). When repeated in 2019 the proportion with hypertension was 27.5%, with 37.5% on treatment and 54.3% with their blood pressure controlled (14). The achieved blood pressure control across centers was comparable to that achieved in Western countries such as USA (46.4% of those treated with blood pressure uncontrolled) (15) and neighboring South Asian countries such as Vietnam (37.7% of those treated with blood pressure uncontrolled) (16). Furthermore, amalgamation potential of telestroke models of care and community-based blood pressure screening programs (4,5) suggests that combined strategies utilizing technology and community-based interventions hold promise for improving stroke management outcomes in South Asia and could form the basis of a stroke management program in the community (17).

To conclude, South Asia’s burgeoning stroke burden demands urgent action. Community based interventions improving blood pressure screening, risk factor management, and rehabilitation are needed to reduce the risk of stroke recurrence and improve quality of life and wellbeing. Collaboration across healthcare, policy, and communities is key to ensuring everyone has access to a healthy life, both before and after stroke.
